# Postoperative ‘STEMI’ in Intracerebral Hemorrhage due to Arteriovenous Malformation: A Case Report and Review of Literature

**DOI:** 10.1155/2019/9048239

**Published:** 2019-04-22

**Authors:** Ravinder Datt Bhanot, Jasleen Kaur, Shitiz Sriwastawa, Kendall Bell, Kushak Suchdev

**Affiliations:** ^1^Department of Internal Medicine, Wayne State University, Detroit, MI, USA; ^2^Division of Pulmonary and Critical Care, St Mary's Of Michigan Medical Center Saginaw, MI, USA; ^3^Department of Neurology, Wayne State University, Detroit, MI, USA

## Abstract

Electrocardiogram (ECG) changes suggestive of cardiac ischemia are frequently demonstrated in patients with ischemic stroke and subarachnoid hemorrhage. However, little is known of such changes particularly acute ST segment myocardial infarction (STEMI) in patients with intracerebral hemorrhage (ICH), especially after neurosurgery. We present a patient with intraparenchymal hemorrhage due to cerebral arteriovenous malformation (AVM) who exhibited acute STEMI after neurosurgery. Serial cardiac biomarkers and echocardiograms were performed which did not reveal any evidence of acute myocardial infarction. The patient was managed conservatively from cardiac stand point with no employment of anticoagulants, antiplatelet therapy, fibrinolytic agents, or angioplasty and recovered well with minimal neurological deficit. This case highlights that diffuse cardiac ischemic signs on the ECG can occur in the setting of an ICH after neurosurgery, potentially posing a difficult diagnostic and management conundrum.

## 1. Background

A number of patients with stroke and aneurysmal subarachnoid hemorrhage (aSAH) have changes on electrocardiogram (ECG) [1]. They can be in the form of T wave inversions, QT interval prolongation, and others. Most of the patients who demonstrate ECG changes also have concomitant heart disease and hypertension which makes it difficult to attribute the ECG changes to the cerebral event alone. Rarely, intracranial hemorrhage (ICH) can also be associated with ECG changes similar to patients with stroke [2]. We present a patient with intraparenchymal hemorrhage due to cerebral arteriovenous malformation (AVM) who exhibited acute ST segment elevation suggestive of myocardial infarction in ECG after neurosurgery. Serial cardiac biomarkers and echocardiograms were performed which did not reveal any evidence of acute coronary event and the ECG changes resolved spontaneously. Acute cerebrovascular events can be associated with fleeting ECG changes or lead to fatal arrhythmias. The mechanism for these changes is poorly understood. Our case represents an uncommon clinical scenario with ICH patients and the challenges in treatment. This case is discussed here for its rarity with a brief review of the literature.

## 2. Case Report

**Figure 1:**
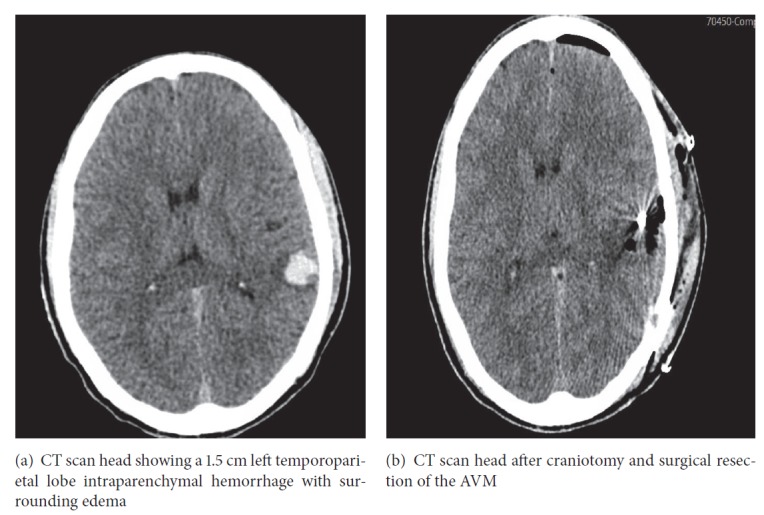


**Figure 2:**
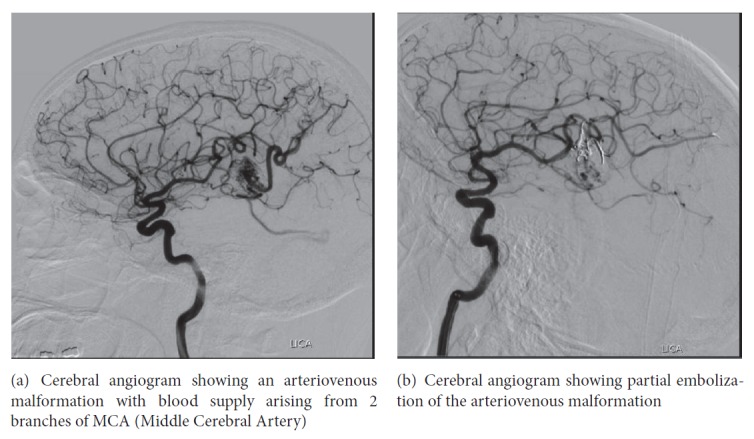


A 42-year-old male, nonsmoker, with medical condition significant for hypertension presented to the emergency department after a fall followed by two episodes of seizures. On presentation physical examination was notable for altered level of consciousness and mild symmetrical decrease in power of 4/5 in all four limbs. Laboratory workup including complete blood count, electrolytes, coagulation panel, lipid profile, urine, and serum drug screen was unremarkable. CT scan head revealed a 1.5 cm left temporoparietal lobe intraparenchymal hemorrhage with surrounding edema as shown in (Figure 1(a)). As part of the diagnostic workup, an ECG was also performed on admission which was normal. The patient was admitted to the neurointensive care unit (NICU) for further management. A computerized tomography angiogram was performed, which showed early draining veins at the site of the lesion, suspicious for an underlying vascular malformation. Subsequently a cerebral angiogram was performed which confirmed the presence of an AVM underlying the hemorrhage (Figure 2(a)). A partial embolization of the AVM was performed, and the patient was boarded for surgical resection (Figure 2(b)).

**Figure 3:**
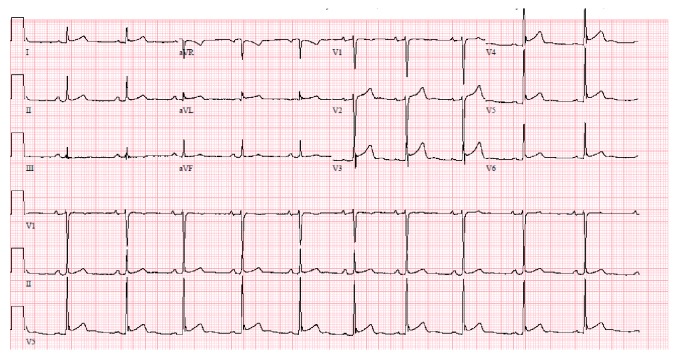
ECG prior to the surgery showing ST segment elevation in leads V3-V6.

**Figure 4:**
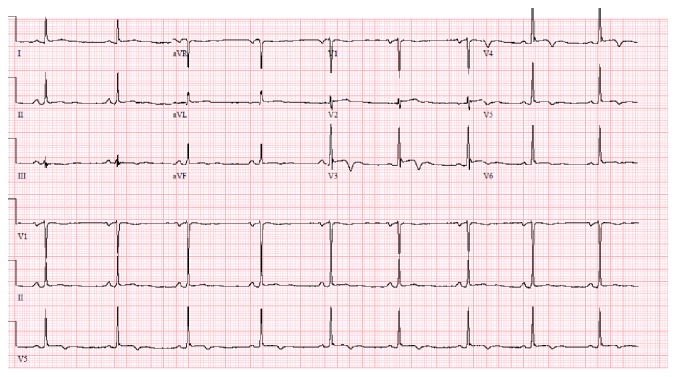
ECG postoperative day 1 showing ST elevation with new T wave inversions in leads V2-V6.

On day 3 of admission, the patient complained of sudden-onset chest pain. He described it as left sided, retrosternal, sharp, nonradiating pain, worsened when lying down on left side, lasted 2-3 minutes and then resolved spontaneously. It did not recur however prompted an ECG which showed sinus rhythm with nonspecific ST segment elevation in leads V3-V6 (Figure 3). Cardiology was consulted who deemed the ECG changes as J point elevation suggestive of benign early repolarization and not a true acute coronary event. A high sensitivity cardiac troponin assay done immediately and repeated two times at 6 hours and 12 hours from the onset of symptoms remained negative (<0.017 ng/ml; normal value <0.057 ng/ml). A transthoracic echocardiogram (TTE) performed later that day revealed no regional wall motion abnormalities or left ventricular dysfunction. The next day, patient was taken for craniotomy and surgical resection of the AVM (Figure 1(b)). The surgery was uneventful. A follow-up ECG on the postoperative day 1 revealed pronounced ST elevation with new T wave inversions (in leads V2-V6) highly suggestive of acute STEMI (Figure 4). The patient was completely asymptomatic with no chest pain or other cardiac symptoms. Serial estimation of high sensitivity cardiac troponin was again negative (<0.017 ng/ml) and a repeat TTE was unremarkable. Given these findings and the absence of the symptoms, no intervention was done and he was monitored in the NICU.

**Figure 5:**
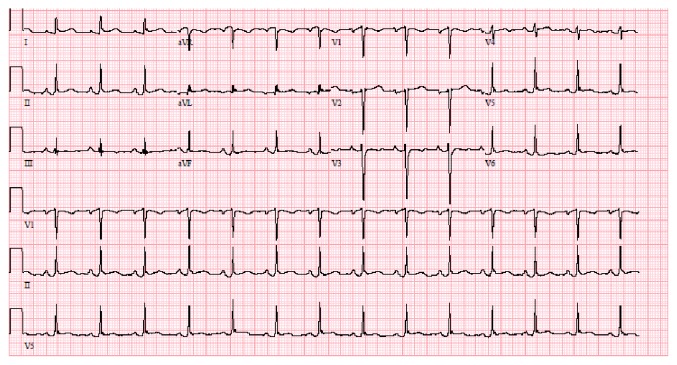
ECG postoperative day 3 showing resolution of ST elevation and T wave inversions in leads V2-V6.

The patient did not have any further untoward event(s) and continued to do well postoperatively with normalization of his ECG changes over the next 48 hours (Figure 5). He improved neurologically and was transferred out of the ICU on day 7. He was subsequently discharged on day 12 with home health physical therapy, neurosurgery, and cardiology follow-up appointments. An exercise stress test was eventually performed 3 months' after discharge which did not reveal any evidence of coronary artery disease.

## 3. Discussion

The electrocardiogram (ECG) is one of the most frequently performed investigations in clinical practice for chest pain. While it is true that an acute myocardial infarction resulting from an occlusive thrombus is recognized on an ECG by ST segment elevation, there are several other conditions and diseases that can simulate or mimic these changes. It can present as a normal finding in some young healthy young men or in a certain pattern commonly referred to as early repolarization. Left bundle branch block, left ventricular hypertrophy, acute pericarditis and myocarditis, stress cardiomyopathy (Takotsubo cardiomyopathy), and certain electrolyte disturbances like hyperkalemia are some of the other nonischemic etiologies of ST elevation. Coronary artery vasospasm can also manifest with indistinguishable ECG changes often warranting a coronary angiogram. Our patient had negative serial cardiac biomarkers and echocardiograms and no significant risk factors other than hypertension (nonsmoker, nondiabetic, normal lipid profile, no cardiac family history, etc.). Hence, a coronary intervention was not deemed necessary and underlying ICH, although not common, was considered responsible for the ECG changes.

ECG abnormalities are commonly seen in association with aSAH and ischemic stroke [3, 4]. Rarely ECG changes and elevated troponins can be seen with ICH. One case series found increased troponins levels in 20% of supratentorial hemorrhages but without confirmatory ECG changes [5]. A recent study by Takeuchi et al. [2] did show that ECG abnormalities can be common in patients with ICH with most frequently observed abnormalities as ST depression (24%) along with T wave inversion and QTc prolongation (both 19%) but not ST elevation (6%) as seen in our patient. Some previous studies also found that these abnormalities particularly QTc prolongation were frequently observed in the acute phase of ICH [6]. However, we did not find any of these or other studies reporting ST elevation in an ICH patient after neurosurgery.

There is some evidence that ECG changes in stroke, including ICH, are caused by over activity of the sympathetic limb of the autonomic nervous system, with insular cortex playing a major in its regulation [7, 8]. These data also indicate some lateralization of cardiovascular representation with sympathetic predominance of cardiovascular regulation being a right insular function, and parasympathetic cardiac neural regulation relating to the left insula [8]. In our patient, the hemorrhage was in the left temporoparietal region involving the left insular cortex. We believe that suppression of left insular outflow likely shifted the autonomic balance towards the right insula and sympathetic predominance. This uninterrupted sympathetic activity may have led to initial ECG changes. These changes became more pronounced with ST elevation and T wave inversion postoperatively due to stress of anesthesia and neurosurgery causing increased levels of circulating catecholamines.

Nonetheless, these ECG findings cause considerable diagnostic problems and management dilemmas in the acute setting. Quite often these ECG changes are benign and self-limiting but lack of information leads to misdiagnosis and unwarranted treatment. This is especially worrisome in patients with ICH because treatment of ischemic heart disease consists of anticoagulants, antiplatelet therapy, or fibrinolytic agents with or without angioplasty which can profoundly worsen the prognosis in these patients [9]. Therefore, sequential evaluation by not only ECG but also serial cardiac biomarkers estimation and echocardiographic assessment is considered necessary for determination of the appropriate therapeutic approach. The same approach was employed in our patient with no untoward effect.

In summary, this case highlights the unique association of an acute cerebrovascular injury and surgical intervention with diffuse ‘ischemic' changes on the ECG. Although the exact reason behind this association is not known, dysregulation of the brain cardiac axis is the probable explanation. Future studies are needed which will help us understand the various unknowns about the brain heart relationship and provide insights into the management of such cases.
